# Recent Advances in Portable and Handheld NIR Spectrometers and Applications in Milk, Cheese and Dairy Powders

**DOI:** 10.3390/foods10102377

**Published:** 2021-10-08

**Authors:** Yuanyuan Pu, Dolores Pérez-Marín, Norah O’Shea, Ana Garrido-Varo

**Affiliations:** 1 Teagasc Food Research Centre, Food Chemistry and Technology Department, Moorepark, Fermoy, Co. Cork, Ireland; Yuanyuan.Pu@teagasc.ie; 2 Department of Animal Production, Faculty of Agriculture & Forestry Engineering, Campus Rabanales, University of Cordoba, Nacional IV-Km 396, 14071 Cordoba, Spain; dcperez@uco.es (D.P.-M.); pa1gavaa@uco.es (A.G.-V.)

**Keywords:** near infrared spectroscopy, handheld sensor, on-site measurement, dairy composition, milk, cheese, powder, authentication issues

## Abstract

Quality and safety monitoring in the dairy industry is required to ensure products meet a high-standard based on legislation and customer requirements. The need for non-destructive, low-cost and user-friendly process analytical technologies, targeted at operators (as the end-users) for routine product inspections is increasing. In recent years, the development and advances in sensing technologies have led to miniaturisation of near infrared (NIR) spectrometers to a new era. The new generation of miniaturised NIR analysers are designed as compact, small and lightweight devices with a low cost, providing a strong capability for on-site or on-farm product measurements. Applying portable and handheld NIR spectrometers in the dairy sector is increasing; however, little information is currently available on these applications and instrument performance. As a result, this review focuses on recent developments of handheld and portable NIR devices and its latest applications in the field of dairy, including chemical composition, on-site quality detection, and safety assurance (i.e., adulteration) in milk, cheese and dairy powders. Comparison of model performance between handheld and bench-top NIR spectrometers is also given. Lastly, challenges of current handheld/portable devices and future trends on implementing these devices in the dairy sector is discussed.

## 1. Introduction

The consumption of dairy products is expected to increase world-wide, particularly in developing countries where there is an increase in population as well as in family incomes [1]. To meet the increasing demand for high quality dairy products, rapid measurements utilised for achieving greater process understanding, quality control and safety assurance are required in every stage of dairy manufacturing, from on-farm milking, to production, and finally to the storage of end-products.

In the past decades, several destructive (i.e., solvent extraction, liquid and gas chromatographic techniques, rheological techniques) and non-destructive (i.e., near infrared, mid-infrared, front face fluorescence spectroscopic techniques) analytical technologies have been implemented in the dairy sector for determination of physicochemical parameters (i.e., protein, fat, viscosity) of various products [2,3,4,5,6,7]. Near infrared spectroscopy (NIRS) technology is one of the most promising spectroscopic analytical technologies in the agri-food field [8], and has gained increasing interest in the dairy industry. Compared to traditional wet-chemistry for routine measurement of chemical composition, the advantages of NIRS technology include its rapid and non-destructive testing, minimal sample preparation, less labour intensive nature and no-chemical consumption [9].

The application of NIRS in the dairy sector dates back to the late 1970s, where it was used to measure low moisture products such as milk powders [10]. Rodriguez-Otero, et al. reviewed the application of NIRS for the determination of major components (i.e., fat, protein, and moisture) of dairy products between 1978 and 1997 [11]. The authors pointed out in the conclusion that the use of fibre optic probes in the production line had great potential in future dairy applications. Giangiacomo and Cattaneo summarised the application of NIRS in different areas along the dairy manufacturing chain, and highlighted some current and potential applications of NIR-based analyses in milk coagulation process, powder authenticity and dairy product classification [10]. A comprehensive review regarding the application of NIR analysis on dairy products was written by Frankhuizen [9,12], providing new updates on the use of NIR to measure major (i.e., moisture, fat, protein, lactose) and minor (i.e., salt, pH, water-soluble primary amines) constituents in different dairy products which included liquid milk, milk powders, casein and caseinates, butter and cheese. Holroyd summarised the application of NIRS in milk and milk products from 2008 to 2012 [13], providing a table with the assignment of important NIR bands for different dairy products (cheese, liquid milk, milk powder). Holroyd also summarised new trends and applications of NIRS in the dairy industry [14], which included understanding the inter-instrument variability, evaluating the performance of the same calibration being used across different locations, and the non-targeted approach for quality assurance. More recently, De Marchi, et al. reviewed the period from 2013 to 2017 [15], focusing on the difficulties to predict chemical components such as fatty acids, minerals and volatile compounds, as well as sensory attributes and ripening time in cheese.

**Figure 1:**
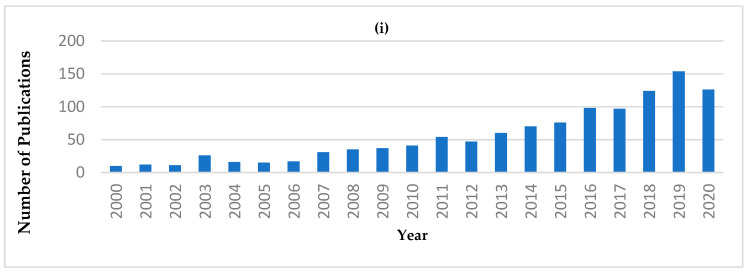

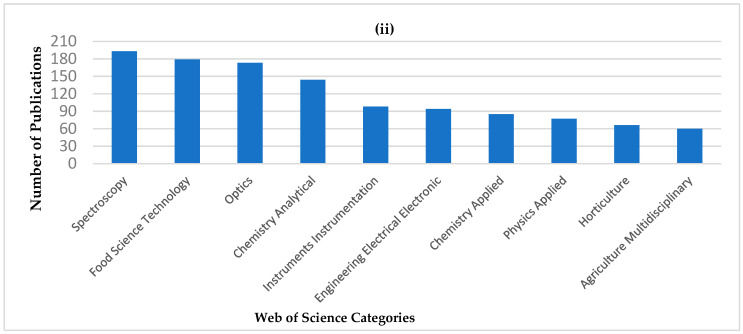
Publication records from the Web of Science database, using the search words “NIR, portable” or “NIR, handheld” or “NIR, hand-held” from 2000 to 2020 (Data accessed on 29 October 2020). (**i**) Number of publications in each year. (**ii**) Number of publications for the first ten Web of Science categories.

From the reviews and publications mentioned above, NIRS has demonstrated its feasibility for the analysis of a variety of dairy products. The number of publications related to “portable or handheld NIR” has shown an increasing trend since 2006 (Figure 1i). Also, the application of portable or handheld NIR in the “Food Science Technology” category has the second greatest number of publication (Figure 1ii). However, within the “Food Science Technology” category, the number of publications regarding to dairy applications is still limited. Publications only begin to appear in 2017, as indicated in Table 1.

To the best of the author’s knowledge, information on the application of portable and handheld NIR analysers in the dairy sector has yet to be reviewed. Therefore, this review aims to provide an overview targeted at dairy processors, researchers, technologists and engineers regarding portable and miniaturised NIR analysers and their potential in the dairy industry. Driven by the concept of “precision dairy farming” and “quality by design (QbD)”, transportable instruments i.e., small versions of laboratory NIR spectrometers [29], are being increasingly used [30,31,32,33]. However, this review, except for comparative purposes, will only focus on small-size and light-weight handheld devices. Firstly, the evolution of handheld or portable NIR devices for agri-food applications is introduced. Secondly, the state-of-the-art of handheld or portable NIR instrumentation reported in literature for dairy applications is briefly described. Thirdly, the applications of NIRS in three major dairy products, i.e., milk, cheese, and dairy powders is reviewed. Lastly, challenges and future trends on the use of handheld and portable NIR devices in the dairy sector is discussed.

## 2. Current Description of Handheld or Portable NIR Devices for Agri-Food Applications

It is outside the scope of this review to describe the current technologies used for portable spectroscopy. Several recent reviews cover in detail the technological developments that have taken place in the last two decades in this area [29,34,35,36,37]. However, in the present review, it is important to briefly mention the main characteristics of the portable spectrometers that have been scientifically evaluated in dairy products (Table 1).

Several portable and on-site NIR instruments have been available since the late 1990s and have been used mainly in the research domain for foods such as fruit and vegetables. However, these instruments did not achieve widespread adoption [38]. At the time, these instruments had some limitations, for example, limited wavelength range (less than 1100 nm), low resolution, poor spectra reproducibility, limited optical window size, the lack of a compartment cell for liquid samples, and the requirement to be connected to an external computer.

Typically bench-top at-line NIR spectrometers are based on optical setups with diffraction grating. Therefore, the main disadvantage for on-site analysis is the presence of mobile parts, their large size and the high price of the bench-top instrument. The strategies to reduce the size of spectrometers are mainly done via the light splitting and detecting components. By the first decade of the 21st century, advances in handheld and micro NIR instrumentation have been made due to the rapid progress on sensing technologies such as Linear Variable Filters (LVF) or micro-electro-mechanical systems (MEMS) and its integration with micro-optics (MOEMS). The precise dimensions and alignment of MEMS devices, combined with the mechanical stability that comes with miniaturisation, make optical MEMS sensors well suited to a variety of challenging measurements [39].

Based on the type of detectors, portable spectrometers can be classified into two categories (array-detector and single-detector instruments). For miniaturised NIR spectrometers, cost and power consumption are major drivers. Therefore, single element detectors are preferred in miniaturised NIR spectrometers; however, the disadvantages of this type of detector is that the spectra obtained are noisier than that obtained by standard InGaAs detectors (with a 1700 nm cut-off), and the detector requires cooling [29,35,40,41]. A summary of the main specifications of handheld or portable NIR devices reported in the literatures is listed in Table 1, including applications in the dairy sector. The following paragraphs will briefly describe the main features of these instruments.

### 2.1. Phazir™ Instrument

In 2005, the Phazir™ handheld NIR spectrometer was launched by Polychromix (now marketed by Thermo Fisher Scientific). This instrument was presented at the 2006 Pittcon conference held in the USA and was claimed to be the first MEMS based handheld NIR spectrometer [42]. Micro-electro-mechanical systems are small parts that integrate electrical and mechanical components into a semiconductor chip for wavelength selection [43,44]. When MEMS is integrated with micro-optics, it is called a micro-opto-electro-mechanical system (MOEMS). These are capable of sensing or manipulating optic signals at a chip-size scale based on the combination of optical, electrical and mechanical technologies [39].

**Figure 2:**
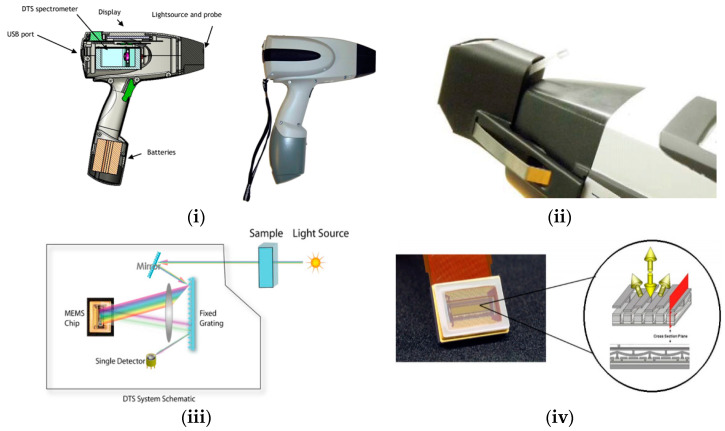
Handheld MEMS Phazir™ spectrometer. (**i**) The sideview of the Phazir™ spectrometer; (**ii**) Microphazir™ with a liquid cuvette adaptor (images are originally from [46]); (**iii**) Schematic of a post-dispersive instrumental design incorporating MEMS technology (image is originally from [46]); (**iv**) MEMS diffraction grating, multi-layer MEMS in reflective state (image is originally from [47,48,49]).

Phazir™ has a programmable MEMS chip, equipped with a fixed diffraction grating and combined with a single detector and a digital transform spectrometer engine, which allows a reduction in size and in the cost of the equipment. This architecture (Figure 2) offers potential advantages over conventional detector-array-based designs, because the use of digital-transform spectroscopy (DTS) not only improves the signal to noise ratio associated with modulation of the light source, but also creates an inherent insensitivity to stray light.

Phazir™ appeared on the market in two different configurations, covering 1100–1700 nm, with a single element InGaAs detector, and a resolution of 6 nm per pixel, or covering 1600–2400 nm (Phazir1624), with a single element InGaAs detector, a resolution of 8 nm per pixel, and with two-stage cooling [29]. Next the MicroPhazir™ was launched, and was only available in the highest wavelength range. MicroPhazir™ consists of the same instrument design as Phazir™, but with some differences, including a smaller size and lighter weight, it contains an internal reference, and it has an information screen to display the operation state of the device [45]. This instrument not only allows the user to scan solids but also liquids using different adaptors (Figure 2ii). Two published papers on dairy products using the MicroPhazir™ were found in the literature (see Table 1). Despite the reduction in the size of the device, this instrument is still heavy (approx. 1.2 kg) for a handheld analyser.

### 2.2. The MicroNIR Spectrometer

**Figure 3:**
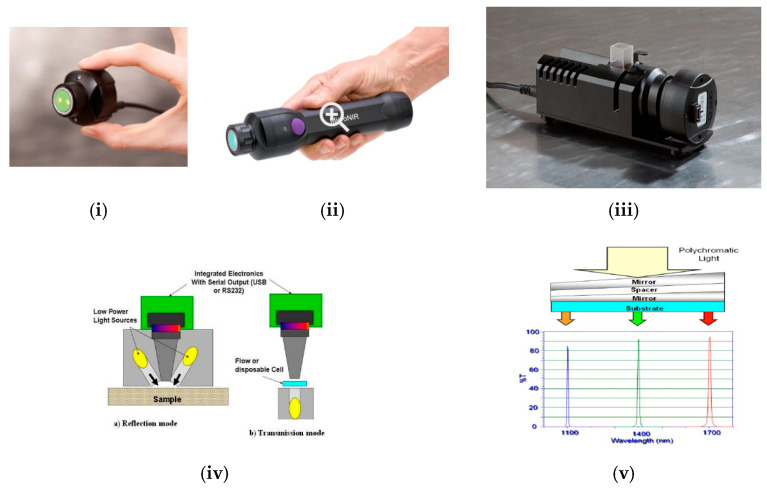
The handheld MicroNIR spectrometer. (**i**,**ii**) The MicroNIR 1700 spectrometer with and without a holder; (**iii**) The MicroNIR Pro and the transmission fixtures; (**iv**) Optical design of MicroNIR working in diffuse reflection and transmission modes; (**v**) Working principle of a linear variable filter (LVF) component. (The images are originally from [50,51,52], copyright permission has been granted).

A pocket-size NIR spectrometer called “MicroNIR” (developed and commercialised previously by JDSU Corporation, Milpitas, CA, USA and currently by VIAVI Solutions Inc., Scottsdale, AZ, USA) was presented at the PittCon^®^ annual conference in 2012 [49]. The MicroNIR spectrometer contains a light source, spectra collection optics, electronics and detector in a package that weighs 58 g and it is powered by a USB connection. The MicroNIR owes its small size and weight to a thin-film linearly variable filter (LVF) technology as the dispersive element, as shown in Figure 3. The LVF is a dielectric thin-film bandpass filter with a coating wedged in one direction. Depending on the thickness of the coating, only light with a specific wavelength can pass through. The coating thickness in a LVF can be customised to select wavelength range on-demand.

MicroNIR was first marketed with two different configurations: covering the standard InGaAs detector wavelength range 950–1650 nm (MicroNIR 1700) as well as the extended InGaAs detector wavelength range 1150–2150 nm (MicroNIR 2200). For both solutions, the MicroNIR incorporates an uncooled detector. Due to power consumption limitations, the cooler alone would have far exceeded the 2.5 W allowable power usage of this USB-powered device [50,51]. A study undertaken by VIAVI Solutions confirmed that the average noise level of the MicroNIR 2200 was 3–5 times higher than that of MicroNIR 1700 [52]. The original and the current MicroNIR spectrometers and their optical design and operating principle are illustrated in Figure 3.

### 2.3. Other Miniaturised NIR Spectrometers

An instrument initially designed for forage analysis at a farm level is the X-NIR™ (Dinamica Generale, Milan, Italy). The NIR unit and the computation unit of the spectrometer are integrated into a single instrument (in total 1.6 kg) which is heavier than MicroPhazir™ (1.2 kg). This instrument can be connected to a computer via USB port or using a WIFI connection, and it is equipped with a touchscreen display for easy access of all functions using fingertips. An application note on using this instrument to predict dry matter, protein and fat content of cheese has been published [23], details are shown in Table 1.

**Figure 4:**
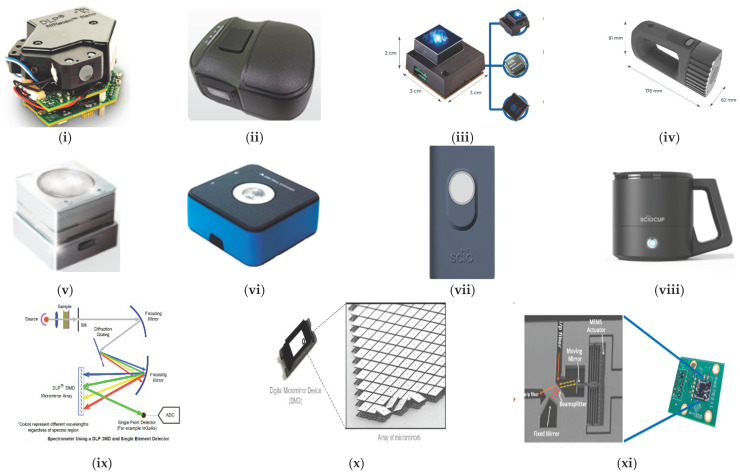
Commercially available handheld NIR devices. (**i**) DLP, the NIR Scan™ Nano optical engine [55]; (**ii**) The NIR-S-G1 sensor based on DLP technology [56]; (**iii**) NeoSpectra Micro monolithic MEMS Michelson Interferometer; and (**iv**) NeoSpectra Scanner [57]; (**v**) NIRONE sensor and (**vi**) NIRONE device (which contains the NIRONE sensor) [58]; (**vii**) SCiO spectrometer and (**viii**) SCiO cup [59]; (**ix**) Spectrometer using a DLP DMD and single element detector; and (**x**) DLP DMD with a close-up of mirror array [54]; (**xi**) Michelson interferometer on a MEMS chip [60]. (All images in Figure 4 are originally from the above cited papers, copyright permission has been granted. Courtesy Texas Instruments for Figure 4i,ix–xi).

Compared to an array detector, the price for a single detector is much lower and in an attempt to further reduce the hardware costs, new developments are focused on systems with single detectors (costs under $5000). Therefore, during the past 5–6 years several companies have launched NIR spectrometers that fit into the palm of a hand [29,36,37,53]. Four of these miniaturised instruments have been used for research in dairy products. They are the NeoSpectra-Micro, NIRONE, Innospectra NIR-S-G1 and SCiO (Table 1). The four NIR spectrometers cover different spectral ranges between 740 nm and 2500 nm and differ in wavelength selection and detection element. Figure 4 provides an image of the instruments, the most recent versions of them, and also provides a schematic view of the optical features.

NIR-S-G1 uses Texas Instruments Digital Light Projector (DLP^®^) Technology. DLP based spectrometers replace the traditional linear array detector with a Digital Micromirror Device (DMD) for wavelength selection and a single point detector [41,53,54]. Figure 4 displays the instruments and the schematic configuration for a DLP and a single detector.

Fourier Transform (FT)-NIR spectrometers provide several advantages over classical dispersive spectrometers such as greater robustness for industrial uses and better resolution and spectra reproducibility. However, miniaturization of a FT-NIR spectrometer is more challenging. It is widely recognised that MEMS technology advances the development of portable NIR instrumentation. The first MEMS FT-NIR sensor, the NeoSpectra, was put onto the market by Si-Ware Systems. This sensor uses a patented MEMS chip (that consists of a Michelson interferometer) and a single element extended InGaAs detector to implement a Fourier Transform approach between 1350 and 2500 nm [29]. Another FT-NIR sensor (The NIRONE Sensor) was launched by Spectral Engines using a similar technology to that used by Si-ware Systems. However, the NIRONE sensor consists of a Fabry-Perot interferometer instead of a Michelson interferometer [58] for the Fourier Transform. Finally, the SCiO (Consumer Physics, Tel-Aviv, Israel) uses a silicon photodiode array (PDA) detector sensitive from around 740 nm to 1070 nm. It was designed to be used by non-NIR-experts and it can be operated using an Android app via Bluetooth [59].

### 2.4. A Note about Software

Most handheld and portable NIR analysers come with software which allows simple data treatments such as the spectra plotted, use of first and second derivative for spectra preprocessing, PLS regression and prediction of unknown samples. However, in order to develop robust models (particularly in high moisture materials), other algorithms (i.e., use of repeatability files, non-linear regression methods, pattern recognition methods, cloning/standardization) are highly recommended.

It should be noted that these software packages allow spectral data to be exported to other chemometrics or multivariate data analysis software (i.e., UNSCRAMBLER, PLS Toolbox, MATLAB, etc.). This is a good option for scientists to explore other methods/algorithms to improve model performance. However, in real applications, a problem arises as one may not be able to import the MATLAB model back into the portable instrument software. In addition, chemometric software i.e., UNSCRAMBLER or MATLAB are expensive software packages, which are not feasible to be included with low-cost portable instruments. Some handheld NIR analyser providers (i.e., SCIO, TELLSPEC) offer cloud computing services, which enables the spectra to be saved in the cloud for data processing later using tools already available on the provider’s website. This cloud-based solution is of limited value for developing a robust model for real world applications. Another problem associated with this type of handheld NIR instrument is the cost to protect the data (i.e., spectral data, reference data, calibration models that latter can be used in a real life application) which can be more expensive than a portable instrument itself. Therefore, data security (i.e., ownership of stored data in the cloud) and data protection (i.e., data protected from being exploited by the handheld NIR analyser providers for use in other applications) should be carefully considered.

Another option offered to consumers is the possibility to predict unknown samples by using the models already incorporated on the app. In the opinion of the authors, this is not advisable as NIRS will always bring up a prediction value, however, it is difficult to know the reliability of the prediction. For example, NIR scientists and practitioners use solid multivariate statistics (i.e., based in Mahalanobis distance) to identify if the spectrum of an unknown sample falls into the calibration population. These type of data quality checks should be included as part of cloud computing services offered by these handheld NIR analyser providers.

## 3. Applications in the Dairy Sector

Applying portable and handheld NIR analysers in the dairy sector is very promising, as they can provide more flexibility to dairy operators for quick quality inspection of the milk supply to final products. The use of portable and handheld NIR devices in the pharmaceutical [61] or agri-food (i.e., fruits and vegetables, meats, feedstock) areas has been previously reviewed [35,44]. In the work of dos Santos, Lopo, Páscoa and Lopes [44], the authors summarised the applications of portable NIR spectrometers in the food industry including dairy product analysis, however only three research studies were mentioned in the review which were related to dairy (milk).

The application of portable and handheld NIR analysers in the dairy sector has become a hot research topic in recent years. Since most of the current portable and handheld NIR devices are not specifically designed for dairy applications, the studies and research available in literature are mainly focused on evaluating NIR performance and exploring its potential applications in the dairy industry.

### 3.1. Milk

Dairy products such as yogurt, cream, cheese and milk powders are made from milk. As a result, raw milk quality and safety play an important role in the production of other milk-based products. This section of the review introduces the use of portable and handheld NIR spectrometers for measuring major and minor chemical constituents of milk as well as to in the detection of milk adulteration.

#### 3.1.1. Major Milk Composition

Kalinin, et al. proposed the use of a portable NIR spectrophotometer (BIKAN-K, weight of 4.6 kg, measuring in transflectance mode in the wavelength range of 800–1080 nm) for the measurement of fat, total protein and lactose in liquid milk [62]. Sixty-five milk samples were prepared by mixing several ingredients (i.e., cream, skim milk, lactose) followed by a homogenisation and a standardisation process, resulting in a milk fat content ranging from 1.5–5.15%, total protein 2.2–4.0% and lactose 4.0–5.8%. Calibration models developed using partial least square regression (PLSR) gave a correlation coefficient of calibration (r^2^_c_) of 0.984, 0.962 and 0.878 for fat, protein and lactose, with a root mean square error for cross validation (RMSECV) of 0.078%, 0.080% and 0.081%, respectively. The authors evaluated the performance of a two-channel portable NIR spectrometer for predicting fat, casein and whey protein in liquid milk [63]. One channel in the spectrometer was used for the acquisition of the transmission spectra and the other channel was used for acquisition of backscattering spectra for each sample. PLSR calibration models based on transmission spectra/backscattering spectra only, or the combination of both were developed. This study demonstrated that prediction models using the combined spectra gave the best result, having a correlation coefficient of prediction (r^2^_p_) of 0.88, 0.89 and 0.91 for fat, casein and whey protein, with a root mean square error of prediction (RMSEP) of 0.08%, 0.13% and 0.07%, respectively.

A handheld MicroPhazir NIR device (Thermo Fisher Scientific., Waltham, MA, USA) was evaluated to develop calibration models for protein, fat and solids-non-fat (SNF) of raw milk [16]. The MicroPhazir spectrometer had a scanning window 4 mm in diameter, which results in a sampling area of 0.13 cm^2^. A liquid adaptor was required for spectral acquisition of liquid milk using this device. Two types of cuvettes, C1 (a reusable quartz cuvette with a 1 mm pathlength for transmission measurement) and C17 (a reusable quartz cuvette with a 17 mm pathlength with an aluminium attached to the other side of the cuvette for transflectance measurement), were assayed in the study to evaluate the spectra quality. The authors confirmed that using the C17 liquid adapter for sampling and setting 80 scans per sample provided the best spectral repeatability and reproducibility. Calibration models based on the modified partial least square (MPLS) method were developed on 482 raw milk samples (444 milk samples as a calibration set and 38 milk samples as an external validation set). The models had a coefficient of determination for calibration (R^2^c) of 0.97, 0.76 and 0.61 for fat, protein and SNF, and a standard error of prediction (SEP) of 0.126%, 0.124% and 0.221%. The authors also evaluated the possibility of transferring the above calibration models to another MicroPhazir device using 10 selected milk samples to build a standardization procedure. Studies demonstrated the capability of sharing calibration data (through a simple calibration transfer procedure) between different handheld MicroPhazir analysers [16].

Four different handheld NIR sensors (NIRONE 1.4, NIRONE 2.0, NIRONE 2.5 in transmission mode and NIRONE 2.0 in reflectance mode, from Spectral Engines, Finland) for measuring fat, protein and lactose of 252 raw milk samples were tested [22]. The PLSR models developed had a prediction error of 0.54–0.58% for fat, 0.30–0.35% for protein and 0.16–0.19% for lactose. The prediction error of NIRONE sensors were compared to international ICAR (The Global Standard for Livestock Data) recommendations for on farm milk analysers (i.e., Tec5 cooled InGaAs spectrometer, AFI milk on-line analyser). The data indicated that NIRONE sensors had a higher prediction accuracy for fat measurement but a lower prediction accuracy for protein and lactose. Amr, et al. investigated the use of a handheld MEMS-based Fourier Transform NIR spectrometer to monitor the fat content in milk [64]. Milk samples with different fat concentrations (0.1%–6.5%) were prepared by mixing skimmed milk with full cream. The spectrometer was operated in transmission mode within a wavelength range of 1300–2500 nm, the milk sample were inserted into a 1 mm pathlength quart cuvette for acquisition of transmission spectra. A calibration model was developed based on the principal component regression (PCR) method, giving a maximum error in concentration prediction of 0.5%. The authors highlighted that the result was acceptable and could be used to determine the categorisation of milk type, i.e., skimmed, low fat, medium fat or full cream milk.

Two miniaturised spectrometers (SCiO and NeoSpectra) were compared for the analysis of commercial milk purchased from Spain, Italy and Switzerland [25]. Both handheld spectrometers could provide a rapid and reliable analysis for the prediction of fat content (in the range of 0.1–3.7%) and milk sample classification (i.e., skimmed, semi-skimmed and whole milk). Since the SCiO (740–1070 nm) and NeoSpectra (1350–2558 nm) cover different spectral regions, a data-fusion PLS model was developed using the spectral data from both sensors, which provided a better fat prediction result than the PLS models developed using each individual sensor.

#### 3.1.2. Minor Milk Composition

Other components in milk, for example, fatty acid profiles, were evaluated using a handheld NIR device (Phazir 1624, 1600–2400 nm) with an opaque liquid cup for spectral acquisition in transflectance mode [21]. Reference data for the fatty acids profile of 108 raw milk samples were determined by gas chromatography mass spectrometry (GC-MS) method. Calibration was carried out by combining spectral pre-treatments and PLSR modelling. Good results were obtained for predicting polyunsaturated fatty acid (PUFA), monounsaturated fatty acid (MUFA), linoleic acid and caproic acid, with a coefficient of determination for prediction (R^2^_p_) that varied between 0.87–0.92, demonstrating the potential of using handheld NIR devices for rapid on-site monitoring of these fatty acids in raw milk at farm level.

#### 3.1.3. Milk Adulteration

Besides chemical composition measurements, handheld NIR analysers were also used to evaluate the capability of detecting milk adulterants. Santos, et al. utilised a handheld NIR device (MicroPhazir, Thermo Fisher Scientific, Waltham, MA, USA) to detect bovine milk (purchased from a local supermarket) and milk spiked with 6 different adulterants (i.e., tap water, whey, hydrogen peroxide, synthetic urine, urea and synthetic milk) at different concentrations ranging from 3 to 50% *v*/*v* [65]. Soft independent modelling of class analogy (SIMCA) method was used to classify the control milk (no adulteration) and adulterated milk samples. It was demonstrated from the results that MicroPhazir provided a correct classification rate (CCR) of 0% for control milk and a CCR of 56% for adulterated milk. These results were compared to a handheld mid infrared (MIR) device (4200 Flex scan, Agilent Technologies Inc., Santa Clara, CA, USA) and a portable MIR system (Cary 630 FTIR spectrometer, Agilent Technologies Inc., USA), showing that both MIR devices provided better performance than the MicroPhazir NIR device (R^2^c = 0.92) for classification and quantification of milk adulterants.

A low-cost digital NIR photometer prototype was proposed by Moreira, et al. for the detection of milk adulteration with water [66]. This prototype was a portable and micro-controlled device, which used three LEDs focused at three wavelengths (970 nm, 1200 nm and 1450 nm); the chosen wavelengths are related to the absorption of water molecules. The diluted milk samples contained 0–25% added water. To measure the percentage of added water, a linear relationship was found between the amplitude of the output signals at each wavelength and the amount of added water, with a mean absolute error of <1%. Liu, et al. compared the performance of a handheld NIR analyser (MicroNIR 1700, VIAVI Solution Inc., Scottsdale, AZ, USA) with a benchtop FT-NIR spectrometer (NIRFlex N-500, Buchi, Flawil, Switzerland) for authentication of organic (37 milk samples) and non-organic (50 milk samples including 36 conventional milks and 14 pasture milks) milk samples bought from a local supermarkets [18]. Partial least square discriminant analysis (PLSDA) method was used for milk classification (organic vs. non-organic). A similar classification rate was obtained using the handheld device (CCR = 73%) and the benchtop instrument (CCR = 78%). Both instruments gave a CCR of 89% for differentiation of the 37 organic and 36 conventional milks. However, based on the fatty acids in the milk samples obtained by the gas chromatography method, the PLSDA classification model had a CCR = 100% to classify organic and non-organic milks.

Similarly, de Lima, et al. investigated the same handheld NIR analyser (MicroNIR 1700) and a benchtop FT-NIR instrument (Perkin Elmer, City, USA) to classify 41 lactose-free ultra-high-temperature (UHT) milk samples (two skimmed milk, 33 semi-skimmed milk and 6 whole milk) with 30 regular UHT milk samples (including six skimmed milk, six semi-skimmed milk and 18 whole milk) [19]. Classification models developed using PLSDA or genetic algorithm linear discriminant analysis (GA-LDA) gave a CCR of 100%, for both the handheld and benchtop NIR instruments, indicating the feasibility of using the ultra-compact handheld NIR device for a quick and precise discrimination between regular and lactose-free milk on-site or in the field.

### 3.2. Cheese

Cheese can be classified as fresh cheese (unripened cheese) and aged cheese (ripened cheese). Most of the cheeses around the world are ripened. During the cheese maturation process, changes in several physical and chemical properties (i.e., composition, pH, texture and flavour) occur [67]. As a result, real-time monitoring of cheese compositions is important to ensure final cheese quality [68].

In recent years reports and publications on the use of handheld NIR analysers in cheese applications have become available. Stocco, et al. compared the accuracy and biases of a portable NIR spectrometer (LabSpec2500, ASD Inc., Boulder, CO, USA) with two different benchtop NIR spectrometers (NIRSystem 5000, FOSS Analytical A/S, Denmark, 1100–2498 nm, measuring in reflectance mode; FoodScan, FOSS Analytical A/S, Denmark, 850–1048 nm, measuring in transmittance mode) for predicting chemical attributes (i.e., dry matter, ash, protein, lipids, water-soluble nitrogen), pH, texture (hardness and working shear force) and the colour of 37 different types of cheese [69]. This research demonstrated that all three NIR instruments had a good prediction performance for the chemical composition, while they were less accurate for pH and texture parameters. The authors also found that the portable one performed better in predicting all the quality attributes compared to the other two benchtop instruments; this is mainly due to the instrument technology of the handheld spectrometer used.

Similarly, three different NIR instruments were evaluated for the determination of fat and dry matter of 160 fresh Swiss cheese blocks (size of 35 × 28 × 12 cm, weight of 12 kg) during the cheese manufacturing process [20]. These instruments were (A) a handheld MicroNIR 1700 (VIAVI Solutions Inc, Scottsdale, AZ, USA) operating in reflectance mode, with a spectral range of 908–1676 nm and a spectral resolution of 6.2 nm, (B) a prototype NIR instrument operating in interaction mode (the light can penetrate in depth down to 1–2 cm), with a spectral range of 760–1040 nm and a spectral resolution of 20 nm, (C) a NIR imaging system (QVision 500, TOMRA Sorting Solutions, Belgium) which collected 15 spectral images from 760 to 1040 nm, with a spectral resolution of 20 nm. Calibration models were developed using PLSR. Results indicated that both NIR instrument (A) and (B) performed equally well for predicting the dry matter content of cheese. However, instrument (A) had a higher prediction accuracy for fat content in comparison to the instrument (B). Instrument (C) had the lowest prediction accuracy for both parameters (dry matter and fat), possibly due to the lower signal-to-noise ratio of the system.

Ma, Babu and Amamcharla evaluated a low-cost handheld NIR device (SCiO, Consumer Physics, Israel) for predicting total protein and intact casein in 49 Cheddar cheeses (35 samples as calibration and 14 samples as validation) using PLSR [28]. The intact casein ranged from 14 to 23 g/100 g of cheese and the total protein ranged from 20 to 25 g/100 g of cheese. Different spectral pre-processing and wavelength selection strategies were applied to improve model performance. Results showed that all models had an RMSEP of 0.91–1.58 g/100 g of cheese for intact casein prediction, while an RMSEP of 0.62–0.88 g/100 g of cheese for total protein prediction. This study indicated that the low-cost SCiO sensor had the potential for rapid and on-site quantification of intact casein and total protein in cheddar cheese. Verena Wiedemair utilised the same NIR sensor (SCiO) to evaluate its performance for the prediction of fat and moisture content of 46 cheese samples [27]. The cheese samples were also grated in order to investigate the impact of cheese physical status (whole pieces versus grated cheese) using a NIR measurement. The same samples were also measured using a benchtop NIR instrument (NIRFlex N-500, Buchi, Flawil, Switzerland) for comparison purposes. Results demonstrated that the PLSR calibration models developed had R^2^_p_ values over 0.93. For fat content, both instruments had a similar prediction accuracy (R^2^_p_ ~0.99 and RMSEP ~0.08%) for grated cheese. However, the SCiO sensor (R^2^_p_ = 0.98, RMSEP = 1.19%) performed better than the benchtop instrument (R^2^_p_ = 0.94, RMSEP = 1.90%) for the prediction of fat in whole pieces of cheese, as the whole pieces of cheese can be spatially inhomogeneous. For moisture content, both instruments gave a similar prediction accuracy for whole cheese, while the benchtop instrument (R^2^_p_ = 0.96, RMSEP = 0.93%) performed better than the SCiO device (R^2^_p_ = 0.93, RMSEP = 1.71%) for the prediction of moisture in grated cheese. Marinoni, et al. purchased 12 portable NIR spectrometers (XNIR, Dinamica Generale, Italy) for evaluating the prediction performance of dry matter (in the range of 64.18–70.16%), fat (in the range of 23.98–32.90%), protein (in the range of 29.29–36.24%), fat to dry matter ratio (in the range of 35.85–48.71), and protein to dry matter ratio (in the range of 43.36–54.20) of Italian hard cheese (Grana Padano) [23]. A total number of 195 slices of protected designation of origin (PDO) Grana Padano cheese that had 6–13 months ripening and were an average weight of 4 kg, were sampled by the consortium from several dairies located in the Po Valley. Samples were scanned with a portable instrument applied directly on the entire slice. After the removal of the rind (first 6 mm from the outside), the cheese was ground and scanned with a benchtop FT-NIR NIRFlex N-500 (Buchi Italia, Cornaredo, Italy). Calibration models were developed using a training set of 116 samples and evaluated with a validation set of 74 samples. The spectra were also acquired at three different temperatures (10, 16 and 25 °C) to minimise the effect of temperature on the model performance. Calibration models based on the cheese paste spectra had a good prediction accuracy for fat, protein, fat to dry matter ratio, and protein to dry matter ratio, with an R^2^_P_ of 0.907, 0.832, 0.902, 0.870 and an RMSEP of 0.461%, 0.396%, 0.602, 0.580. The results were comparable to those obtained with a benchtop FT-NIR instrument (RMSEP = 0.54%, 0.49%, 0.67, 0.47 for fat, protein, fat-to-dry matter ratio, protein-to-dry matter ratio). However, the PLSR model yielded a relatively poor performance for dry matter (R^2^_p_ = 0.616 and RMSEP = 0.654%), possibly because there is a moisture gradient inside the cheese which affects the sample homogeneity that could have an impact on the performance of the calibration model. Predictive models based on the cheese rind spectra were built for fat to dry matter ratio and protein to dry matter ratio, which had a R^2^_P_ value close to 0.6. Nevertheless, this study proved the use of small and cost-effective portable NIR devices for predicting cheese composition at batch level, which is important for small scale factories who produce Grana Padano cheese.

### 3.3. Dairy Powders

Dairy powders including skimmed milk powder, whole milk powder and milk/whey protein powders can be used as ingredients in baking, recombined food products and infant formula [70]. The quality and safety of dairy powders should be monitored on a regular basis to ensure they have a consistent composition and functionality and meet a high safety standard, especially for infant formula production. The NIRS technology in dairy powders is mainly used for compositional measurements and powder adulteration detection.

#### 3.3.1. Powder Composition

Kalinin et al. applied a portable NIR device (BIKAN-CP, equipped with a fibre-optic and heat-resistant probe) to measure moisture, fat and protein of a dried milk mixture [62]. The NIR device operated in reflectance mode and in the wavelength region of 1050–1670 nm. The optimum PLSR models obtained had a correlation coefficient value of 0.945, 0.981, 0.842 and a RMSECV value of 0.25%, 1.87% and 3.62% for moisture, protein and fat, respectively. Another type of portable NIR instrument (FieldSpec Pro, Analytical Spectral Devices, Inc., Boulder, CO, USA) was studied by Wu, et al. [71], to investigate its performance for the simultaneously measurement of fat, protein and carbohydrate in 350 infant milk powder samples, using the short-wave NIR with the spectral region of 800–1050 nm. Spectral pre-treatments combined with PLSR or least squares-support vector machine (LS-SVM) were employed for model development. The models yielded good prediction accuracy, with an R^2^_p_ of 0.98 achieved for the three components. The authors also investigated the wavelength assignments for fat, protein and carbohydrates based on the loading weights and regression coefficients derived from the models. For example, the wavelength 861 nm and 897 nm were assigned to the third overtone of C-H stretching vibration of carbohydrate; the wavelength 968 nm was assigned to the second overtone of O-H stretching present in water; and the wavelength of 1033 nm was assigned to the second overtone of N-H stretching present in fat.

Kong, et al. utilised the same NIR device (FieldSpec Pro) but using different wavelength regions (350–1075 nm) to detect irradiation doses of irradiated milk powders, as irradiation technology are commonly used to destroy microorganisms in foods [72]. However, the use of gamma-irradiation may have an effect on protein functionality due to possible oxidation of amino acids and formation of protein free radicals. A total of 150 milk powders were irradiated with gamma-rays at different doses (0–6 kGy) and stored in a controlled environment (temperature = 25 ± 1 °C, humidity = 70%) for seven days prior to the NIR spectra being taken. The best model obtained in the study had a correlation coefficient of 0.97 and an RMSEP of 0.844 kGy, indicating the feasibility of portable NIR devices in quantification of irradiation dose in milk powders.

#### 3.3.2. Powder Adulteration

Studies on evaluating the use and sensitivity of NIRS integrated with chemometrics for rapid determination of melamine and other possible adulterants in milk powders has increased since the milk scandal incident that occurred in China in 2008 [73,74,75].

Henn, et al. compared two miniaturised NIR spectrometers, a MicroNIR 2200 (from VIAVI Solutions, Scottsdale, AZ, USA) and a MicroPhazir (from Thermo Fisher Scientific, Waltham, MA, USA) with a benchtop NIR instrument (NIRFlex N-500, Buchi, Flawil, Switzerland) for melamine detection in milk powder (infant formula) [17]. The adulterated milk powders were prepared by adding pure melamine (purity ≥ 99%) into off-the-shelf infant formula powders, to reach a final adulteration concentration from 0% to 5.5% which was added at 0.5% increments. The best PLSR calibration model achieved by each instrument had a similar RMSEP value of 0.28 g/100 g (melamine/milk powder), 0.33 g/100 g and 0.27 g/100 g for the NIRFlex N-500, the MicroPhazir and the MicroNIR 2200 instrument. The authors further calculated the limit of detection (LOD) interval [LOD_min_, LOD_max_] for the three spectrometers using the calculation proposed by Allegrini and Olivieri [76]. The three instruments had a LOD interval of [0.20, 0.30] g/100g, [0.28, 0.54] g/100 g and [0.44, 1.15] g/100 g, indicating that the benchtop instrument was more sensitive for detecting low concentrations of components like melamine, and the benchtop spectrometer had a wider wavelength region and a more sophisticated instrument design. The authors highlighted that additional information (i.e., the LOD interval) should be taken into consideration when evaluating the robustness of models.

Karunathilaka, et al. investigated two benchtop FT-NIR spectrometers (from the manufacturers Bruker and PerkinElmer) and a handheld NIR device (Phazir 1624, Polychromix Inc., Wilmington, MA, USA) for non-targeted detection of contaminations in commercial non-fat-dried-milk powders [77]. Eleven potential adulterants were considered in the study, which can be categorised into four groups: (1) low molecular weight, nitrogen-rich compounds (i.e., melamine); (2) plant-based proteins (i.e., soy protein); (3) inorganic salts (i.e., calcium carbonate); and (4) non-fat solids (i.e., sucrose). Classification models based on the SIMCA method demonstrated that the benchtop FT-NIR instruments yielded a higher sensitivity and specificity for authentication of milk powder when compared to the portable device, as the portable device had a narrower spectral range and a lower spectral resolution.

Zinia Zaukuu, et al. compared a benchtop NIR spectrometer (MetriNIR, Development and Service Co., Hungary, wavelength region of 750–1700 nm with a spectral resolution of 2 nm) and a handheld spectrometer (NIR-S-G1, InnoSpectra Co., Taiwan, wavelength region of 700–1700 nm with a spectral resolution of 3 nm) to detect low concentration of nitrogen-based adulterants in whey protein powder [26]. Using four different adulterants, urea (U), melamine (M), glycine (G) and taurine (T), 15 types of adulterated whey protein powders were prepared, by adding one single adulterant (U, G, T, M), two adulterants (GT, GU, GM, TU, TM, UM) or more than two adulterants (GTU, GTM, GUM, TUM, GTUM) to the whey protein powders. The final adulteration level was from 0.5 to 3 (%, *w/w*) with an increment of 0.5%. Three sets of NIR spectra were acquired, dataset 1 was obtained by placing the powder samples in an optical glass cuvette and scanned by the benchtop instrument; dataset 2 was obtained by placing the powder samples in an optical glass cuvette and scanned using the handheld device; dataset 3 was obtained by placing powder samples in a low density polyethylene zip-lock bag and scanned using the handheld device. PLSR was used to quantify the adulteration concentration and linear discriminant analysis (LDA) was used to classify adulterants in different scenarios. Overall, the benchtop NIR instrument performed the best in predicting these nitrogen-based adulterants. The handheld instrument was not sensitive in detecting the lowest concentration (0.5%). However, for other concentrations, reliable results were achieved, proving the advantages of handheld devices for on-site quality screening of whey protein powders though glass cuvettes or plastic bags.

## 4. Comparison between Handheld and Benchtop Instruments

There are many factors which affect NIR model performance. Williams mentioned three different error sources [78], that is, those related to the instrument (i.e., signal-to-nose-ratio, wavelength accuracy), sample (i.e., granulometry, affected by temperature fluctuations) and operational (i.e., sample preparation, high errors in the reference data, chemometric method used). Therefore, one of the difficulties when evaluating portable instruments, is the correct interpretation of model performance, in particular, what are the reasons or factors that cause the differences in the models between portable and benchtop instruments.

Some authors have tried to overcome this by developing in parallel (on a portable instrument and on a benchtop instrument) predictive models with identical samples. In the case of dairy products, most studies have been done using a low number of samples and without proper validation. As a result, the interpretation of the results is sometimes very optimistic, and therefore some publications suggest that portable devices have a similar performance to benchtop instruments.

Table 2 summarises the research applications where a comparison between both portable and benchtop instruments were carried out using dairy products. Liu, et al. [18] and de Lima, et al. [19] compared the MicroNIR 1700 with two different bench top instruments, to evaluate the ability to qualitatively analyse liquid milk, for two different authentication issues (organic vs. non-organic, lactose free vs. regular milk containing lactose). Both studies concluded that the models developed with the MicroNIR 1700 have a CCR similar to those obtained with the benchtop instruments. However, in both cases the sample type (bought from the market) and the low number of samples used make it difficult to believe that the models developed are sufficiently robust to lead to this this conclusion. For cheese studies, researchers found that the MicroNIR 1700 and the SCiO portable devices had a similar prediction accuracy to the NIR-Flex bench top instrument for predicting macro composition (i.e., dry matter, protein, fat) of several types of cheese [20,23,27]. According to the studies reported on milk powders, current handheld devices are not as sensitive as benchtop NIR instruments in detecting adulterants in milk powders in terms of prediction accuracy [17,26,77]. It is also worth noting that based on the experimental design presented in the mentioned studies, it is not possible to form firm conclusions as not all the information is given for a more rigorous analysis of the data.

## 5. Challenges and Future Trends

Portable and handheld NIR analysers have gained increasing attention in the dairy industry as a potential at-line process monitoring tool to be used on site or alongside the processing line for fast product screening. Miniaturised NIR devices can offer many benefits to dairy producers in their routine product checks. Nevertheless, the handheld NIR devices are more of a complementary tool rather than a replacement of benchtop spectrometers.

It is clear that portable, handheld and miniaturised devices are under continuous development and it is critical that the number of scientific publications related to the evaluation of new instruments increases. However, these studies should be undertaken following standardised evaluation protocols which at least consider the minimum number of samples to use, the evaluation of the spectral repeatability, noise levels, stability of the optical readings in different environmental conditions, optimisation of sample presentation (especially in the case of liquids and semi-liquids), and proper validation of the developed models.

One of the challenges for the current handheld NIR instruments is the sensitivity of the measurements and the applicability. The potential applications of miniaturised NIR devices can be overly simplified by the suppliers of the technology. As well, the developed NIR models require ongoing model maintenance, which is a detail that is neglected to be communicated to the customer. For example, the application models advertised on the supplier website for agri-food products (e.g., fruit) are not robust enough to model the wide variability that can exist in the chemical composition of such products, as well as the influence of the environment and production factors. It should be mentioned that the software used for rapid data analysis is also required. Some handheld NIR analyser providers offer a cloud-based solution for easy access, storage and analysis of NIR data. However, caution should be taken regarding cloud connectivity, data safety and data protection. In the upcoming years, there will be a growth in the use of open-source tools, such as R and Python and computation in the cloud. This will allow researchers of portable instruments to develop their own calibration and classification models, as well as bespoke apps for mobile/tablet devices for a given product/application.

Despite this, there is an obligation to contribute to the body of knowledge within NIR spectroscopy regarding the potential of these novel NIRS spectrophotometers. It can also act as the object (front end) sensing technology as part of an Internet of Things (IoT) network. Without a doubt, these compact, economical, and miniaturised spectrometers already represent a new era in NIRS technology.

## Figures and Tables

**Table 1:** Specifications of handheld or portable NIR devices reported in literature with applications in the dairy sector.

Commercial Name	Manufacturer	Wavelength (nm)	Measurement Mode	Light Source	Wavelength Selector	Detector	Weight and Size	Dairy Applications	Reference
MicroPhazir	Thermo Fisher Scientific Inc. (USA)	1600–2400	Reflectance Transmittance	Tungsten	MEMS	InGaAs	Weight: 1.2 kg	Liquid Milk (raw milk from individual cows)	[16]
MicroNIR 2200	VIAVI Solutions Inc. (USA), formerly known as JDS Uniphase Corporation, USA	1128–2162	Reflectance	Two tungsten lamps	LVF	InGaAs	Weight: <60 g	Milk powder	[17]
MicroNIR 1700/1700ES	VIAVI Solutions Inc. (USA), formerly known as JDS Uniphase Corporation, USA	950–1650	Reflectance; Transmittance; Transflectance	Two integrated vacuum tungsten lamps	LVF	InGaAs	Weight: 64 g Size: 45 × 50 mm	Liquid milk (pasteurized retail milks, UHT milks)	[18,19]
								Cheese	[20]
Phazir 1624	Polychromix Inc. (USA), sold to Thermo Fisher Scientific in 2010	1600–2400	Reflectance	-	MEMS	InGaAs	1.7 kg	Liquid Milk (raw milk from individual cows)	[21]
NIRONE	Spectral Engines (Finland)	1100–2500	Transmittance	Two tungsten vacuum lamps	MEMS	InGaAs	Weight: 15 g; Size: 25 × 25 × 17.5 mm	Liquid milk (raw milk from individual cows)	[22]
X-NIR	Dinamica Generale	950–1800	Reflectance	-		-	Weight: 1.6 kg;	Cheese	[23]
NeoSpectra	Si-Ware Systems (Egypt/Europe/ USA)	1350–2500	Reflectance	Three lamps	MEMS	photodetector	Spectral resolution:16 nm; Weight: 17 g; Size: 32 × 32 × 22 mm	Liquid Milk (raw milk from individual cows; commercial UHT milks)	[24,25]
NIR-S-G1	InnoSpectra Co., (Taiwan)	750–1700	Reflectance	Tungsten-Halogen	Based on Texas Instruments DLP technology	InGaAs	Weight: 87 g; Size: 76 × 82 × 27 mm	Milk powder	[26]
SCiO	Consumer Physics (Israel)	740–1070	Reflectance	-	-	-	Weight: 35 g; Size: 3.15 × 9.5 × 27.5 mm	Cheese	[25,27,28]
								Liquid milk (commercial UHT milks)	

**Table 2:** Comparison of model performance between handheld/portable NIR instruments and benchtop NIR instruments in the dairy products.

Reference	Research Objective, Predictive Models, Number and Type of Samples	Handheld/Portable NIR Spectrometer			Benchtop NIR Spectrometer		
		Instrument	Multivariate Model	Performance	Instrument	Multivariate Model	Performance
[18]	Organic milk authentication, N = 37 organic milk and N = 50 non-organic retail milks	MicroNIR 1700 (VIAVI Solution Inc., Scottsdale, AZ, USA) 908–1670 nm	PLS-DA	CCR = 73%	NIRFlex N-500 (Buchi, Flawil, Switzerland) 1000–2500 nm	PLS-DA	CCR = 78%
[19]	Lactose-free milk authentication, N = 30 regular UHT milk samples and N = 41 lactose-free UHT milks	MicroNIR 1700 (VIAVI Solution Inc., Scottsdale, AZ, USA) 908–1670 nm	PLS-DA	CCR = 100%	FT-NIR Spectrum Frontier (Perkin Elmer, USA) 833–2500 nm	PLS-DA	CCR = 100%
			SPA-LDA	CCR = 80%		SPA-LDA	CCR = 100%
			GL-LDA	CCR = 100%		GL-LDA	CCR = 100%
[69]	Moisture, protein, lipids; N = 197 cheese samples	LabSpec2500 (ASD Inc., USA) 350–1830 nm	Bayesian regression (whole spectral range,350–1830 nm)	Moisture: R^2^_CV_ = 0.96 RMSECV = 2.10%	NIRsystems5000 (FOSS, Denmark) 1100–2498 nm, in reflectance; grinding the sample	Bayesian regression (whole spectral range, 1100–2498 nm)	Moisture: R^2^_CV_ = 0.83 RMSECV = 4.51%
				Protein: R^2^_CV_ = 0.88 RMSECV = 1.77%			Protein: R^2^_CV_ = 0.81 RMSECV = 2.25%
				Lipids: R^2^_CV_ = 0.85 RMSECV = 2.03%			Lipids: R^2^_CV_ = 0.67 RMSECV = 3.20%
			Bayesian regression (common spectral range,1100–1830 nm)	Moisture: R^2^_CV_ = 0.96 RMSECV = 2.00%		Bayesian regression (common spectral range, 1100–1830 nm)	Moisture: R^2^_CV_ = 0.84 RMSECV = 4.39%
				Protein: R^2^_CV_ = 0.91 RMSECV = 1.59%			Protein: R^2^_CV_ = 0.76 RMSECV = 2.51%
				Lipids: R^2^_CV_ = 0.85 RMSECV = 2.03%			Lipids: R^2^_CV_ = 0.69 RMSECV = 3.11%
		LabSpec2500 (ASD Inc., USA) 350–1830 nm	Bayesian regression (whole spectral range,350–1830 nm)	Moisture: R^2^_CV_ = 0.96 RMSECV = 2.10%	FoodScan (FOSS, Denmark) 850–1048 nm, in transmittance; grinding the sample	Bayesian regression (whole spectral range, 350–1830 nm)	Moisture: R^2^_CV_ = 0.83 RMSECV = 4.51%
				Protein: R^2^_CV_ = 0.88 RMSECV = 1.77%			Protein: R^2^_CV_ = 0.81 RMSECV = 2.25%
				Lipids: R^2^_CV_ = 0.85 RMSECV = 2.03%			Lipids: R^2^_CV_ = 0.67 RMSECV = 3.20%
			Bayesian regression (common spectral range,850–1050 nm)	Moisture: R^2^_CV_ = 0.96 RMSECV = 2.30%		Bayesian regression (common spectral range,850–1050 nm)	Moisture: R^2^_CV_ = 0.77 RMSECV = 5.26%
				Protein: R^2^_CV_ = 0.91 RMSECV = 1.57%			Protein: R^2^_CV_ = 0.73 RMSECV = 2.62%
				Lipids: R^2^_CV_ = 0.85 RMSECV = 2.03%			Lipids: R^2^_CV_ = 0.64 RMSECV = 3.28%
[27]	Moisture and fat, N = 46 cheese samples (whole and grated)	SCiO (Consumer Physics, Israel); 740–1070 nm	PLSR (on whole cheese)	Moisture: R^2^_P_ = 0.94 RMSEP = 1.14%	NIRFlex N-500 (Buchi, Flawil, Switzerland); 1000–2500 nm	PLSR (on whole cheese)	Moisture: R^2^_P_ = 0.94 RMSEP = 1.10%
				Fat: R^2^_P_ = 0.98 RMSEP = 1.19%			Fat: R^2^_P_ = 0.94 RMSEP = 1.90%
			PLSR (on grated cheese)	Moisture: R^2^_P_ = 0.93 RMSEP = 1.71%		PLSR (on grated cheese)	Moisture: R^2^_P_ = 0.96 RMSEP = 0.93%
				Fat: R^2^_P_ = 0.99 RMSEP = 0.82%			Fat: R^2^_P_ = 0.99 RMSEP = 0.77%
[23]	Dry matter, fat and protein; N = 195 Grana Padano cheese	X-NIR (Dinamica Generale Electronic Solutions & Sensors, Italy) 950–1800 nm	PLSR (on grinded cheese pasture)	Dry matter: R^2^_P_ = 0.62 RMSEP = 0.65%	NIRFlex N-500 (Buchi, Flawil, Switzerland); 1000–2500 nm	PLSR (on grinded cheese pasture)	Dry matter: R^2^_P_ = N/A RMSEP = 0.71%
				Fat: R^2^_P_ = 0.91 RMSEP = 0.46%			Fat: R^2^_P_ = N/A RMSEP = 0.54%
				Protein: R^2^_P_ = 0.83 RMSEP = 0.40%			Protein: R^2^_P_ = N/A RMSEP = 0.49%
[17]	Melamine detection; N = 111 milk powder samples	MicroNIR2200 (VIAVI Solution Inc., Scottsdale, AZ, USA); 1128–2162 nm	PLSR	R^2^_P_ = 0.96 RMSEP = 0.27%	NIRFlex N-500 (Buchi, Flawil, Switzerland); 1000–2500 nm	PLSR	R^2^_P_ = 0.96 RMSEP = 0.28%
		MicroPhazir (Thermo Fisher Scientific, Waltham, MA, USA); 1600–2400	PLSR	R^2^_P_ = 0.95 RMSEP = 0.33%			
[77]	Non-targeted milk powder authentication; (N > 67)	Phazir 1624 (Thermo Fisher Scientific, Waltham, MA, USA) 1596–2400 nm	SIMCA	The authors highlighted hat portable device of limited utility for non-targeted detection of adulteration of this food commodity	FT-NIR (Bruker Multi Purpose Analyser (MPA), USA) 800–2500 nm	SIMCA	CCR = 100% for Melamine, Aminothiazole, Biuret at a 0.6–2% adulteration level
					FT-NIR (Perkin Elmer, USA) 1000–2500 nm		
[26]	Whey protein powders authentication; (N = 819)	NIR-S-G1 (InnoSpectra Co., Taiwan); 750–1700 nm	PLSR (common spectral range 950–1650 nm)	Urea: R^2^_P_ = 0.91 RMSEP = 0.25%	MetriNIR (MetriNIR Research, Development and Service Co., Hungary); 950–1650 nm	PLSR (common spectral range 950–1650 nm)	Urea: R^2^_P_ = 0.92 RMSEP = 0.23%
				Glycine: R^2^_P_ = 0.75 RMSEP = 1.03%			Glycine: R^2^_P_ = 0.85 RMSEP = 0.82%
				Taurine: R^2^_P_ = 0.85 RMSEP = 1.37%			Taurine: R^2^_P_ = 0.90 RMSEP = 1.14%
				Melamine: R^2^_P_ = 0.82 RMSEP = 0.53%			Melamine: R^2^_P_ = 0.86 RMSEP = 0.21%

CCR = correct classification rate.

## Data Availability

No new data were created or analyzed in this study. Data sharing is not applicable to this article.
